# A Transdisciplinary Approach to Managing Emerging and Resurging Mosquito-Borne Diseases in the Western Pacific Region

**DOI:** 10.3390/tropicalmed2010001

**Published:** 2017-01-06

**Authors:** Margaret C. Hardy, Dani J. Barrington

**Affiliations:** 1 Division of Chemistry and Structural Biology, Institute for Molecular Bioscience, The University of Queensland, St Lucia, Brisbane, QLD 4072, Australia; 2 Department of Marketing, Monash University, Clayton, VIC 3800, Australia; 3 International Water Centre, Brisbane, QLD 4000, Australia; 4 School of Public Health and Global Change Institute, The University of Queensland, Brisbane, QLD 4072, Australia

**Keywords:** citizen science, mosquitoes, vector-borne disease, participatory action, WaSH, Zika, Western Pacific

## Abstract

Mosquitoes transmit a number of harmful diseases that have an impact on local communities and visitors, and many pose a threat to neighboring countries. As federal monitoring budgets shrink across the world, the increasing importance of citizen scientists in monitoring and identifying invasive species, as well as acting to prevent these diseases, are discussed. Examples of past mosquito management programs are provided, and future directions are discussed with an emphasis on the Western Pacific Region.

## 1. Introduction

### 1.1. Mosquito-Borne Diseases in the Western Pacific Region

The World Health Organization defines the globe in terms of six geographical regions. This includes the Western Pacific Region (WPR), which consists of 37 countries and approximately one quarter of the Earth’s population, with many of the countries being classified as less developed [1]. Many of the countries in the WPR are island nations, physically separated from one another, and even islands of the same country, by the Pacific Ocean and various seas. Mosquito-borne diseases proliferate in the WPR [2], posing a health risk to local communities and visitors. Because the WPR is more physically connected than ever (e.g., diseases can be transported by boat or plane, in the form of either the infected insect vector or infected host [3]), mosquito-borne diseases also pose a risk to those residing in more developed countries in the region, particularly Australia and New Zealand.

The pest and public health risks associated with mosquitoes are a major concern for communities in the WPR. In the WPR, several mosquito-borne disease pathogens are vectored by a phylogenetically diverse range of mosquitoes (Table 1). As well as being home to several genera of mosquitoes which vector different mosquito-borne disease pathogens, the incidence of mosquito species varies throughout the region. Malaria is one of the most well-studied mosquito-borne diseases worldwide, and provides a case study of the complexity of vector-borne diseases in the WPR. The specific emphasis is on illustrating that the WPR is not a monolith. According to the WHO, 735 million people in the Western Pacific Region are at risk of contracting malaria, with 31 million being at high risk; there were an estimated 812,000 malaria cases in the region in 2014 [4]. Malaria is clearly an issue of concern in the WPR but the key vector and parasite species differ across the 46 countries (Table 2). As such, it is important that the prevention and control of mosquito-borne diseases is based upon local species and conditions; programs for the prevention and control of mosquito-borne diseases cannot consider the WPR as a monolith [5].

Endemic dengue fever caused major epidemics every 10 to 40 years in the early 20^th^ century throughout the WPR, but World War II dramatically changed the landscape for dengue fever and likely resulted in the hyperendemic dengue fever now found in many cities of the WPR [9]. Another mosquito-borne pathogen of interest to the region is the Zika virus, which has been reported in several WPR countries: Cambodia, Cook Islands, Federated States of Micronesia, Fiji, French Polynesia, Marshall Islands, New Caledonia, Philippines, Samoa, American Samoa, Solomon Islands, Tonga, and Vanuatu [10,11]. Zika virus is a flavivirus transmitted by *Aedes* mosquitoes that was first discovered in a sentinel monkey in 1947 in Uganda, and soon after in humans in Nigeria. The virus was mainly restricted to the African continent until the 1980s, when it spread to Southeast Asia, and from there to Micronesia in 2007. After reaching the Americas in 2014, the virus has undergone a rapid spread that can best be described as “explosive” [12]. The history of the spread of Zika virus has been shown to resemble that of dengue and chikungunya viruses [13]. This illustrates that a peri-urban cycle of emerging arboviruses could be a risk to countries across the WPR, which share a similar climate. Further, the particular challenges of managing an outbreak of a complex vector-borne disease with nonspecific symptoms are amplified in the WPR, where the public health infrastructure to manage disease outbreaks is widely variable both between countries and within islands of a single country.

Mosquito-borne disease outbreaks have health effects on humans and an impact on the economy. For example, concurrent outbreaks of dengue, chikungunya, and Zika in the WPR are believed to have impacted productive work hours [2]. Even where reported cases of mosquito-borne outbreaks are low, travel advisories suggesting certain viruses may be present can also impact on visitor numbers, economically damaging the tourism industry [14].

### 1.2. Biosecurity

As climate change may increase global temperatures, the potential spatial range of arthropod species able to vector diseases may also increase [15,16,17]. Combined with subsequent increases in travel and urbanization, this means more individuals are likely to come into contact with mosquito-borne diseases specifically [3]. Biosecurity is defined by the National Academy of Sciences as “security against the inadvertent, inappropriate, or intentional malicious or malevolent use of potentially dangerous biological agents or biotechnology, including the development, production, stockpiling, or use of biological weapons as well as natural outbreaks of newly emergent and epidemic diseases” [18] and is key to preventing the spread of pathogens across country borders. Ectoparasites such as mosquitoes are of biosecurity concern if they have three characteristics: (1) arrival: the ectoparasite is able to survive the quarantine measures (if any) in place before, during, and after transit; (2) establishment: the ectoparasite is able to find a suitable habitat to colonize in the new location; and, (3) spread: the ectoparasite can adapt to life cycle differences in the new location, like the availability of suitable hosts and/or breeding sites. The flow of mosquito-borne pathogens across international borders due to poor biosecurity further widens their threat to human health.

### 1.3. Water-Related Diseases

**Figure 1:**
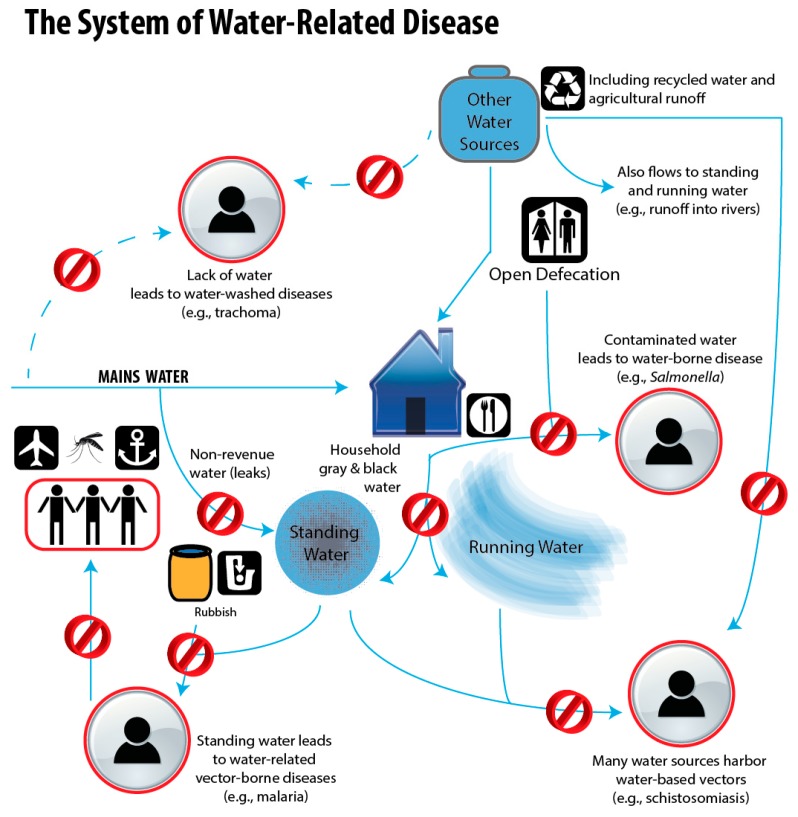
The system of water-related disease. The red circle with the line through (the prohibition symbol, Ø) indicates areas where efforts to reduce pathogen transmission are most effectively focused.

Since mosquitoes require water to breed, they are part of the complex of diseases that fall into the “water-related” classification system [19]. Improper water treatment, transport, drainage systems, solid waste disposal and hygiene behaviors (from hereon referred to as “improper water, sanitation and hygiene (WaSH)”) contribute to poor environmental health conditions where water-related diseases proliferate (Figure 1). Standing water that collects naturally in some areas, or results from anthropogenic sources like leaking pipes, wastewater overflows, and rainwater collection in solid waste, offers ideal breeding sites for container- and some wetland-breeding mosquitoes [20,21,22]. For example, in Laos and Thailand, a positive association was identified between poor water storage in areas affected by diarrheal disease and an increase in dengue transmission [23]. In the Philippines, a study determined a positive association between poor solid waste disposal and cases of chikungunya [24].

Urban population growth is now outstripping national growth in the WPR [25,26,27]. This is resulting in densely populated urban and peri-urban settlements, where improper WaSH is common and diseases caused by poor environmental health are rife [28]. Increased urbanization has been linked to an increase in dengue cases across the globe, including countries of the WPR [29]. Without controlling mosquito populations, the transmission of pathogens can be prevented in some cases, most famously through the use of insecticide-treated or untreated bednets for night-biting mosquitoes, such as *Anopheles*, which vector the malaria protozoa [30]. However, where bednets are not used, not effective, or mosquitoes bite during the day or early evening, measures to understand and control mosquito populations are vital in reducing the spread of such diseases. Many mosquito genera could be controlled to a greater extent with improved WaSH [31,32,33].

### 1.4. Insecticides: the Silver Bullet?

Insecticides are compounds used to kill insects, and vary widely in their origin. Insecticide sources can be biological, botanical, bacterial, or chemical, and with recent advances in formulation and separation chemistry, the search for novel sources of sustainable insecticides has accelerated in recent years [34]. Early chemistries relied on broad-spectrum toxins that had unanticipated impacts on the environment and non-target species—including in some cases humans, livestock, or native wildlife. Recently, the number of chemicals that can be used in integrated pest management programs in tandem with biological and cultural control programs has increased the safety, efficacy, and longevity of insecticides [35]. Chemical insecticides are a keystone component of many management programs for vectors of human diseases, including in the use of insecticide-impregnated bednets to prevent night-biting mosquitoes spreading human pathogens like malaria.

In addition to concerns about environmental and non-target impacts, the main pressure on conventional chemical insecticides is the development of insecticide resistance across a range of active ingredient classes [36]. Insecticide resistance evolves by two main mechanisms in arthropods: the increased production of detoxifying enzymes, or mutation of the target with subsequent decreases in sensitivity [37,38].

Conceptually, insecticide resistance relies on two components: the number and type of insects that become resistant to an active ingredient; and the number and type of active ingredients that have demonstrated insecticide resistance. The Insecticide Resistance Action Committee (IRAC, http://www.irac-online.org) monitors the target site of insecticides and its mechanism of action (MOA) classification tool is designed to help design appropriate rotations to manage resistance management in chemical control. Although 28 insecticide classes are currently recognized based on their differing MOAs (plus one additional class of insecticides with unknown MOAs), the number of molecular targets for the different chemistries are far fewer. Despite recent efforts to better manage the development of resistance across novel MOAs, insecticides that target acetylcholinesterase, chloride, and sodium channels make up 90% of the cases of resistant species and 65% of the active ingredients to which resistance has developed [36]. The development of insecticide resistance in mosquitoes or other vectors of disease should not be separated from the development of resistance in agricultural or other contexts, because the development of insecticide resistance in a local region is connected to the other management programs taking place simultaneously [34].

Aside from the reduced effectiveness of insecticide treatments, there are increasing concerns about their environmental sustainability in controlling mosquito populations, especially as traditional chemical control methods have an impact on invertebrates that play a critical role in sustainable agriculture and in other ecological processes, like pollination [35,39]. Perhaps most critically, insecticide resistance renders many of our safest and most potent chemicals ineffective, and this is particularly true for the four insecticide classes approved for use in mosquito control by the WHO [36]. Because the active ingredients in the four WHO-approved insecticide classes hit the same two molecular targets, the discovery of novel insecticide targets is key for continued mosquito management [34,40,41,42].

### 1.5. A New Mosquito-Borne Pathogen Management Approach for the Western Pacific Region

Historically, the WPR has been a hotspot for mosquito-borne disease management. The management method for dengue fever during the post-WWII period was control of the principal urban vector, *Aedes aegypti*. Although dengue fever was never eradicated from the region, control of the mosquito vector enabled the level of pathogen transmission to dip before a resurgence in the 1970s and 1980s because of the development of insecticide resistance by the mosquito vector [43,44]. Levels of demographic and societal changes not recorded previously in human history are now aiding the spread of pathogens and mosquitoes, and areas without robust public health infrastructure, such as less developed countries, are likely to face additional challenges in management [2].

Decision makers in the WPR recognize that climate change will increase the problems due to vector-borne pathogens and water insecurity, particularly for the island states [45], and that because many of the species responsible for the spread of human pathogens exhibit widespread insecticide resistance [41], chemical control methods alone are unlikely to provide adequate management.

With the challenges of climate change, urbanization, and improper WaSH, as well as the increasing potential for pathogens to be transferred across international borders, we suggest that a systems-based approach to preventing mosquito-borne diseases in the WPR will be critical to addressing the issue from multiple angles. Systems thinking is about linking qualitative and quantitative information across disciplines, without resorting to reductionism, so as to understand a situation as an entire system, rather than tackling only its component parts [46]. We must consider not only reducing the physical transmission of these pathogens, but also the infrastructural, behavioral, and agricultural factors that contribute to their proliferation. The spread of these diseases will not be curbed by high level policies and procedures alone, but must be tackled through a holistic systems-based approach to improving environmental health that includes a consideration of local context and the contribution that local communities can make to reducing disease prevalence [43,47].

## 2. Exploring Mosquito and Pathogen Populations through Citizen Science

Understanding the different kinds of insect vectors and their preferred breeding habitat can help manage the incidence of mosquito-borne pathogen transmission. For example, Japanese encephalitis is a mosquito-borne pathogen of concern in the WPR, and research from China has demonstrated that temperature, rainfall, and humidity are predictors of the abundance of mosquitoes that vector the disease. Understanding such factors can assist in developing risk management programs to reduce the transmission of pathogens to and between humans [48]. Several online invasive species databases have been closed in the past decade; of particular interest to the WPR are PaDIL, Hawaiian Ecosystems at Risk (HEAR), the Pacific Basin Information Node (PBIN), and Pacific Ecosystems at Risk. This is unfortunate, given the progress made with online decision-making tools for an integrated approach to ectoparasite management, as seen with the success of the ParaBoss suite of products [49].

Citizen science programs recruit members of the public to assist in the collection of large datasets of interest [50]. Such programs are generally either cross-sectional (e.g., how many bird species are in one area) or longitudinal (e.g., how many birds breed in one area each year) in their design [51]. For example, a number of citizen science programs have emerged to help identify and measure the spread of invasive organisms [52], such as invasive plants [53], ants [54], and marine species [55]. Citizen science initiatives in the WPR are proving useful following the closure of online databases, with websites like BowerBird (http://www.bowerbird.org.au/) providing online space for experts and individuals to share biological sighting information and collaboratively identify animals in photographs. Community members are responsible for being the first to spot the red imported fire ant (*Solenosis invicta*) in Queensland in 2001, as well as in 2015, finding the first report of *Miomantis caffra*, a South African mantid that had been recorded in New Zealand but not Australia [56]. A citizen scientist initiative in Papua New Guinea has focused on using mobile phones to collect data on disease outbreaks that can be used to monitor their spatial distribution and assist in allocating resources [57]. Although citizen scientists should not be expected to correctly identify mosquitoes in the WPR correctly to the species level—a task beyond many except the most seasoned entomologists—ecological or behavioral data (e.g., collection location and time of biting) provide a wealth of useful information to help plan future directions for monitoring and management programs.

As these approaches become more widely used, giving communities the science literacy skills and tools to identify disease symptoms, insects, and other invasive species will be critical, and sustained outreach and engagement programs will play an important role in ensuring the integrity of community-led data collection [58]. The training of citizen scientists and collection of data on mosquito species prevalence, alongside factors which contribute to it (e.g., WaSH infrastructure, community behaviors, and quantitative data on the presence of stagnant water) could usefully contribute to developing management programs to prevent mosquito-borne pathogen spread in the WPR.

## 3. A Systems-Based Approach to Reducing Mosquito-Borne Disease

### 3.1. The “One Health” Approach

Taking a systems-based approach to “‘wicked problems’ wherein stakeholders may have conflicting interpretations of the problem and the science behind it, as well as different values, goals, and life experiences” allows practitioners to address issues in a transdisciplinary context which can contribute to improving issues around environmental health [59]. The One Health approach is an emerging systems-based paradigm where human health, animal health and environmental professionals work together in a transdisciplinary model to achieve better outcomes more efficiently [60]. To be successful at preventing mosquito-borne diseases, stakeholders must be engaged from line ministries to communities themselves, and in national policy to individual behavior change.

Using the One Health approach to improve WaSH may reduce the spread of mosquito-borne pathogens without reliance on insecticides. There has been a tendency for WaSH to be siloed into the sub-topics of drinking water, toilets, handwashing, solid waste management, and menstrual hygiene, and separated from the larger water cycle [61]. However, as Figure 1 demonstrates, mosquito-borne diseases are part of a large system of WaSH provision and treatment, where both large scale failures (e.g., city-wide non-revenue water) and individual behaviors (e.g., improper disposal of solid waste) can have negative health impacts on the wider community, including members who do have access to appropriate water and sanitation. For example, a household may have proper water pipes and wastewater conveyance systems, and practice hygienic behaviors, but if they reside in an area where mosquito breeding is prevalent in stagnant water collecting in errant solid waste then it is entirely possible that they too are at high risk of mosquito-borne diseases.

Furthermore, the system within which mosquito-borne diseases reside is not purely technical or behavioral. It includes many exchanges of products and services, social capital, and monetary capital for communities and individuals to meet their needs. In-depth research in informal settlements of Fiji, Solomon Islands, and Vanuatu has demonstrated the importance of human relationships, income, aesthetics, and education to individual and household acquisition of WaSH products and services [33,62]. Anthropogenic land use and water redirection for non-household purposes also contribute by causing a cascade of factors that increase the potential for infectious diseases to emerge, including deforestation, increased pathogen movement, environmental contamination, poverty, and immigration [63]. The One Health approach to pest management can also slow the development of insecticide resistance, by only applying conventional chemical insecticides when necessary. This is made explicit in policy guidelines, as evidenced by the WHO’s approval of only four classes of insecticides for controlling the mosquito vector of malaria [64].

Taking a One Health approach to the problem of mosquito-based pathogen transmission in the WPR will allow for policy makers and practitioners to address the issue at varying levels, within different contexts, and whilst considering transmission factors beyond simply improving physical infrastructure or screening infected persons at air and sea ports.

### 3.2. Integrating the One Health Approach into Citizen Science Programs

Although citizen science is typically used for the collection of large data sets, such participatory methods of data collection can also be used at the community level to empower action. Approaches which combine participatory monitoring and action view the practical problems of the stakeholders and study communities as the starting point of investigation, and assume that the potential solutions also reside in local knowledge [65]. Coupling participatory monitoring to participatory action allows for nuanced, locally responsive investigations of mosquito-borne disease [66]. It can also incorporate the One Health approach to managing systems by integrating studies based on citizen science data with behavior change, education, and environmental improvements.

An example of this approach to data collection and action is the WPR’s Locally-Managed Marine Area (LMMA) Network (http://www.lmmanetwork.org/). Formally established in 2000, the LMMA Network brings together community-managed biodiversity projects which are developed based upon local cultural practices and beliefs and focus on both community monitoring and action [67]. It has seen particular success in Fiji where communities have monitored and managed coral reef ecosystems, including adapting community behaviors to those which will conserve the environment. There has been strong support from the Fiji federal government to implement these community-management strategies, to the extent that the Fiji LMMA Network has influenced policy and mainstreamed community-management into government fisheries practice [68].

In the Solomon Islands success has been seen in using a participatory action research (PAR) approach to collect health data and implement change. Members of the Atoifi Health Research Group have been working with Atoifi Adventist Hospital for over a decade, engaging local community members and hospital staff to collect and analyze data, and develop and implement action plans. One project developed culturally appropriate health facilities for community members of various ethnic backgrounds [69]. The Research Group also has a current project which builds on its earlier work, to engage local communities in biodiversity conservation for the improvement of health, clearly echoing the One Health approach to system-based action.

Both the LMMA Network and Atoifi Health Research Group have used locally appropriate methods of participation to empower community members to improve their well-being through self-determined action. The success of these programs suggests that engaging community members in citizen science and action is a reality in the WPR, which could be adapted to controlling mosquito populations through improving WaSH and other behaviors.

### 3.3. Integrating the One Health Approach into Biosecurity Measures

Although participatory monitoring and action can manage local populations, it is important to recognize that it is not fail-safe, and practitioners need to look more broadly at biosecurity. An increased appreciation for the global, cosmopolitan nature of vectors of biosecurity concern and both the large (e.g., continental) and small (e.g., community) systems within which they operate must evolve. Implementing an area-wide One Health approach that crosses political boundaries would improve our ability to monitor and prevent the spread of mosquito-borne pathogens.

Unlike early programs that relied only on chemical control for the eradication of disease vectors, newer biosecurity programs are focused on prevention and integrated pest management. The One Health approach that believes human, animal, and environmental health to be interconnected is a useful framework for approaching management programs. With container-breeding mosquitoes in particular, by managing environmental and animal health appropriately we effectively decrease breeding grounds and habitat for disease vectors. Eradication may not be practical due to cost, environmental concerns, insecticide resistance, or other non-target impacts, but with human disease eradication must always be the goal. Particularly with sensitive ecosystems like islands, an integrated approach that considers chemical, cultural, or behavioral, and biological control options is preferred [70]. There are suitable chemical control options that can be used in tandem with biological controls, but the programs should be carefully designed in advance [35]. These mixed-method area-wide management programs are often the only effective approach for ectoparasites [71]. Area-wide programs that cross political boundaries and rely on multiple methods of pest management from a One Health approach are highly effective, as was the case in a number of previous programs (Table 3). The key feature of all the programs is coordination and effective communication across a range of stakeholders.

**Figure 2:**
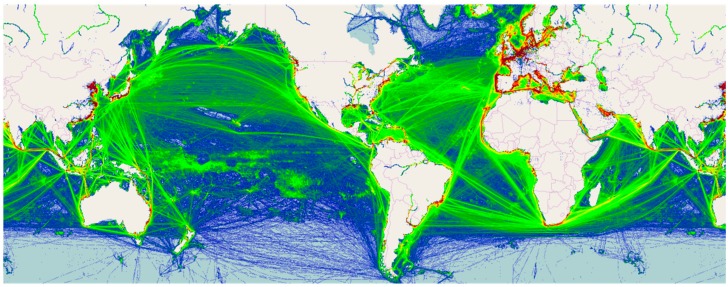
Global marine traffic in 2014 (map used with permission from MarineTraffic [78]). The color-coding is based on a compound algorithm, and refers to the number of distinct vessels on a daily basis per square km: blue (<30), green (30–70), yellow (71–140), and red (>140).

Just as communities cannot be expected to eliminate mosquito populations on their own, individual governments cannot rely solely on their own ability to identify and treat incursions, and regional and trade partners should focus on collaborative efforts to manage pest incursions along direct trade (Figure 2) and tourism routes. This is particularly true for insects in the Pacific Region, where the origin of invasive species is often unknown or difficult to determine [78]. Incorporating data sets collected by citizen scientists at all scales can provide a much larger perspective than traditional research projects, in both number and geographic range covered, and can provide those with expertise in biostatistics open-access datasets to analyze.

## 4. Conclusions

An integrated One Health approach to the prevention of mosquito-borne pathogens in the WPR could be implemented from the community to the regional level. Although data collection by research professionals of course remains important, monitoring and analysis by citizen scientists can contribute to larger scale pest management whilst concurrently empowering communities to enact their own changes to reduce disease spread at the local level.

## Figures and Tables

**Table 1:** Several of the most medically important vectors of mosquito-borne diseases present in the Western Pacific Region, and a selection of diseases each mosquito species transmits in the region.

Genus	Bites During	Vectors
*Aedes* spp. (esp. *A. aegypti*, *A. albopictus*)	Daytime	Chikungunya, dengue, Zika (also heartworm in animals)
*Anopheles* spp.	Nighttime	Malaria, filariasis
*Culex quinquefasciatus*	Nighttime	Filariasis

**Table 2:** Malaria parasite profiles of countries in the Western Pacific Region. Data is from the World Malaria Report, 2015 [6].

Country	Major *Plasmodium* Species	Major *Anopheles* Species	Reported Confirmed Cases/Deaths
Cambodia	*P. falciparum* (64%), *P. vivax* (36%)	*An. dirus*, *An. minimus*, *An. macultus*, *An. sundaicus*	25,152/18
China	*P. falciparum* (11%), *P. vivax* (88%)	*An. sinensis*, *An. anthropophagus*, *An. dirus*, *An. minimus*	2921/24
Lao People’s Democratic Republic	*P. falciparum* (62%), *P. vivax* (38%)	*An. dirus*, *An. minimus*, *An. maculatus*, *An. jeyporiensis*	48,071/4
Malaysia ^a^	*P. falciparum* (7%), *P. vivax* (8%)	*An. balabacensis*, *An. donaldi*, *An. maculatus*, *An. sundaicus*, *An. flavirostris*	3923/9
Papua New Guinea	*P. falciparum* (56%), *P. vivax* (41%)	*An. farauti*, *An. punctulatus*, *An. koliensis*	281,182/203
Philippines	*P. falciparum* (81%), *P. vivax* (17%)	*An. flavirostris*, *An. maculatus*, *An. balabacensis*, *An. litoralis*	4903/10
Republic of Korea	*P. vivax* (100%)	*An. sinensis*	638/0
Solomon Islands	*P. falciparum* (54%), *P. vivax* (46%)	*An. farauti*, *An. punctulatus*, *An. koliensis*	18,404/23
Vanuatu	*P. falciparum* (12%), *P. vivax* (88%)	*An. farauti*	982/0
Viet Nam	*P. falciparum* (54%), *P. vivax* (46%)	*An. minimus*, *An. dirus*, *An. sundaicus*	15,752/6
**Total**			**401,928/297**

^a^ In Malaysia, the major *Plasmodium* species of concern is *P. knowlesi* [7,8].

**Table 3:** Examples of successful area-wide arthropod pest management programs.

Pest	Program Region	Reference
Codling moth	USA	[72]
Fruit fly	Hawai`i	[73]
Screwworm fly	The Americas	[74]
Termites	SE USA	[75]
*Aedes* mosquitoes	Australia	[76]
Blacklegged and lone star ticks	NE USA	[77]
Tsetse flies	Senegal	[78]
